# Supplementary material for: Primary-like Human Hepatocytes Genetically Engineered to Obtain Proliferation Competence as a Capable Application for Energy Metabolism Experiments in In Vitro Oncologic Liver Models
Source: Biology (Basel). 2022 Aug 9;11(8):1195. doi: 10.3390/biology11081195 (PMC9405410; doi:10.3390/biology11081195)
Supplement: Supplementary file 1 [file biology-11-01195-s001.zip › biology-1786009-supplementary.pdf]

Supplementary material

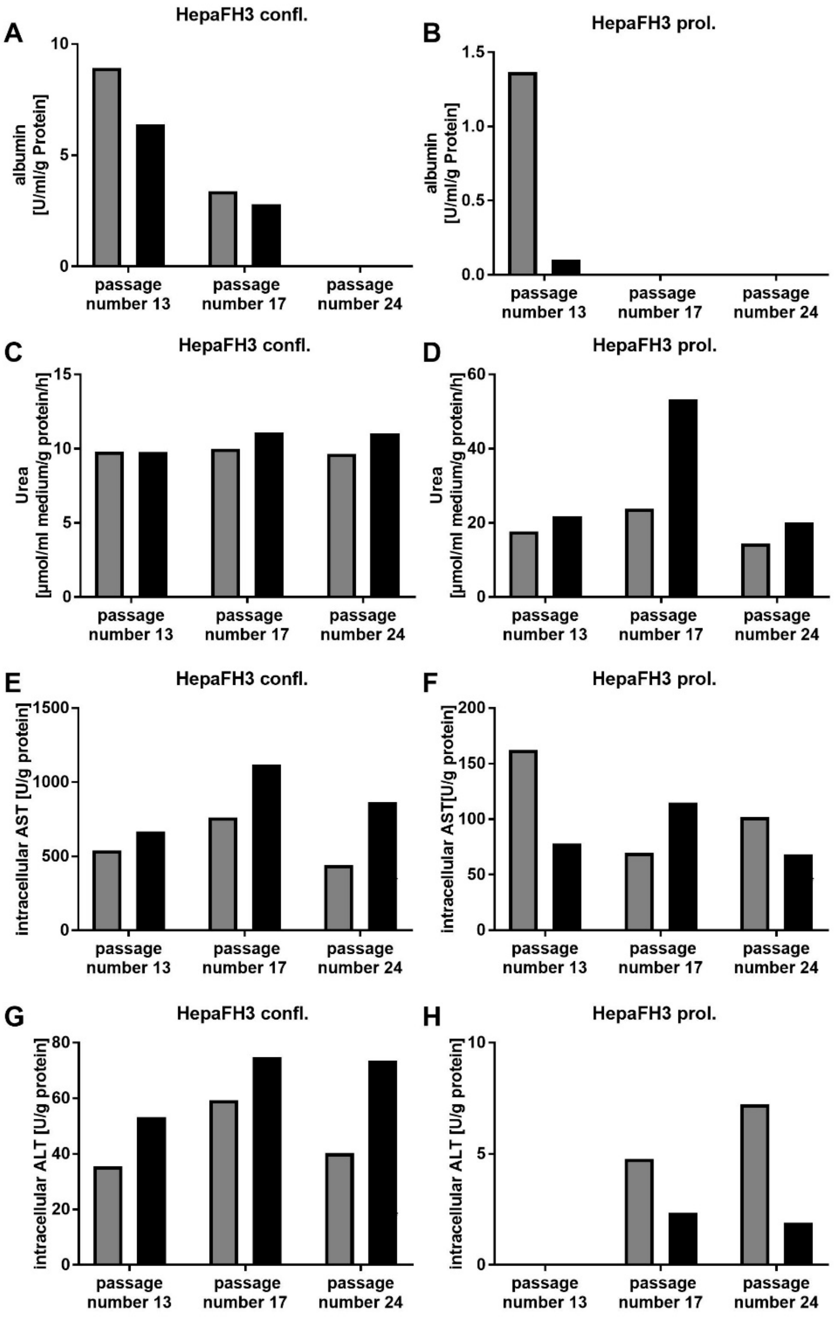

Figure S1 = Basic hepatocyte markers and their dependence on cell passage number of HepaFH3 cells. Albumin **(A, B)**, Urea **(C, D)**, intracellular AST **(E, F)** and intracellular ALT **(G, H)** for different cell passage numbers of differentiated and proliferating HepaFH3 cells is given. Confl. = confluent, Prol. = proliferating

A

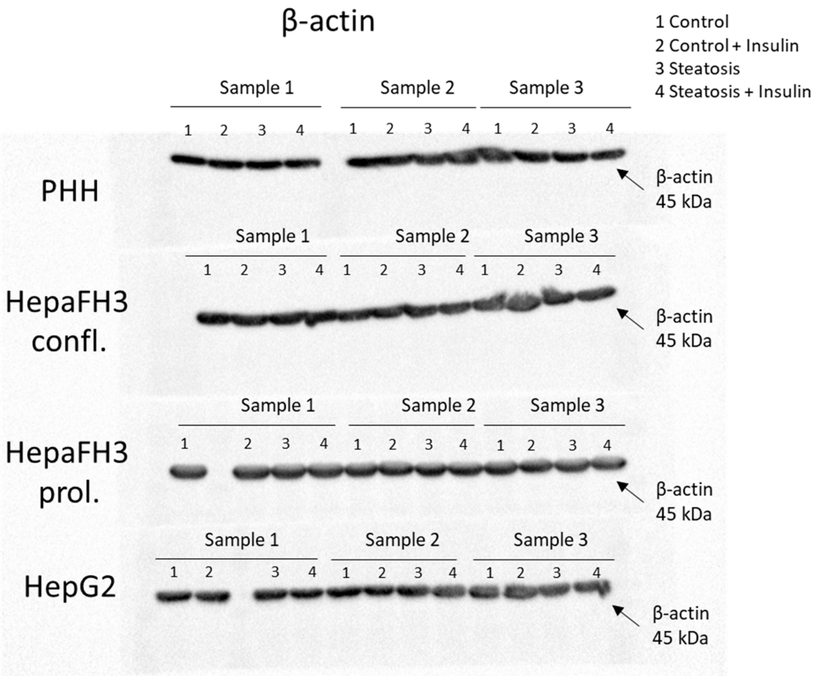

B

		densitometric readings β-actin			
		**PHH**	**HepaFH3 confl.**	**HepaFH3 prol.**	**HepG2**
		**DR-Blank**	**DR-Blank**	**DR -Blank**	**DR - Blank**
Sample 1	1	5580876	6190054	6249012	5173504
	2	5454512	5587218	6510339	5367124
	3	5722752	5981846	6400020	5451264
	4	5044172	6345040	6214725	5126828
Sample 2	1	5849984	5184816	6243864	5775196
	2	5594736	5488210	6457671	5191508
	3	5421332	5478096	6190008	5558700
	4	6721288	4810234	6189579	5035100
Sample 3	1	6494068	5690776	6284157	5226620
	2	5854184	6362850	6003591	5541676
	3	5404840	5871476	5595909	4605440
	4	4619104	5075954	5457672	5358472

Figure S2 = Western Blot analysis of β-actin (45 kDa) for PHHs, HepaFH3 confl., HepaFH3 prol. and HepG2 cells. (A) Cell lysates of the different cell types have been separated by SDS-PAGE and blotted on separate membranes. For all cell types, experiments have been performed in triplicates. **(B)** Densitometric readings (DR) for every band is depicted. Blank of an empty line has been deducted from the DR. Exposure time = 17 sec for all blots. PageRuler™ Prestained Protein Ladder, 10-180 kDa from Thermo Fisher was used as protein ladder. Confl. = confluent, Prol. = proliferating, DR = densitometric reading.

A

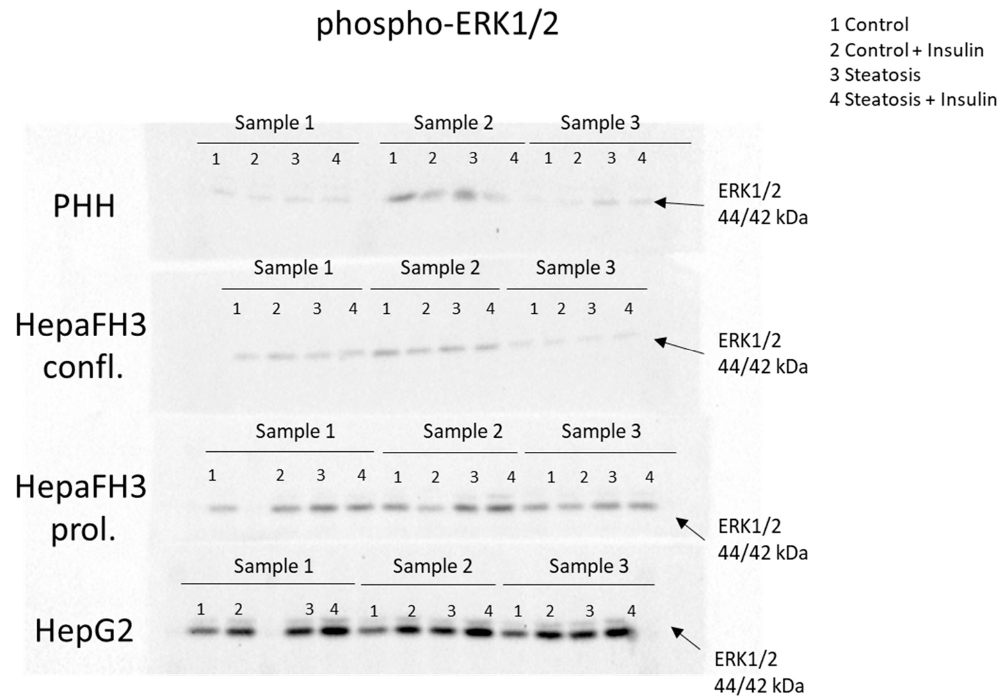

B

		densitometric readings pERK			
		**PHH**	**HepaFH3 confl.**	**HepaFH3 prol.**	**HepG2**
		DR-Blank	DR-Blank	DR -Blank	DR -Blank
Sample 1	1	393680	530504	660786	1004402
	2	294210	1010061	1166116	1598940
	3	398188	774150	1613472	1701336
	4	362530	926263	1419264	2558374
Sample 2	1	2143736	1658592	1245426	1103984
	2	1361388	1080001	563388	1802794
	3	2206106	1413633	1575896	1557262
	4	786758	1184222	2438590	2604448
Sample 3	1	65226	409097	1017982	1136310
	2	157612	293111	791392	1921332
	3	711662	212849	1359512	1747788
	4	295498	314301	1278158	2430316

Figure S3 = Western Blot analysis of pERK1/2 (44/42 kDa) for PHHs, HepaFH3 confl., HepaFH3 prol. and HepG2 cells. (A) Cell lysates of the different cell types have been separated by SDS-PAGE and blotted on separate membranes. For all cell types, experiments have been performed in triplicates. **(B)** Densitometric readings (DR) for every band is depicted. Blank of an empty line has been deducted from the DR. Exposure time = 335.5 sec for PHHs, 416.9 sec for HepaFH3 confl., 213.4 sec for HepaFH3 prol., 91.4 sec for HepG2. PageRuler™ Prestained Protein Ladder, 10-180 kDa from Thermo Fisher was used as protein ladder. Confl. = confluent, Prol. = proliferating, DR = densitometric reading.

A

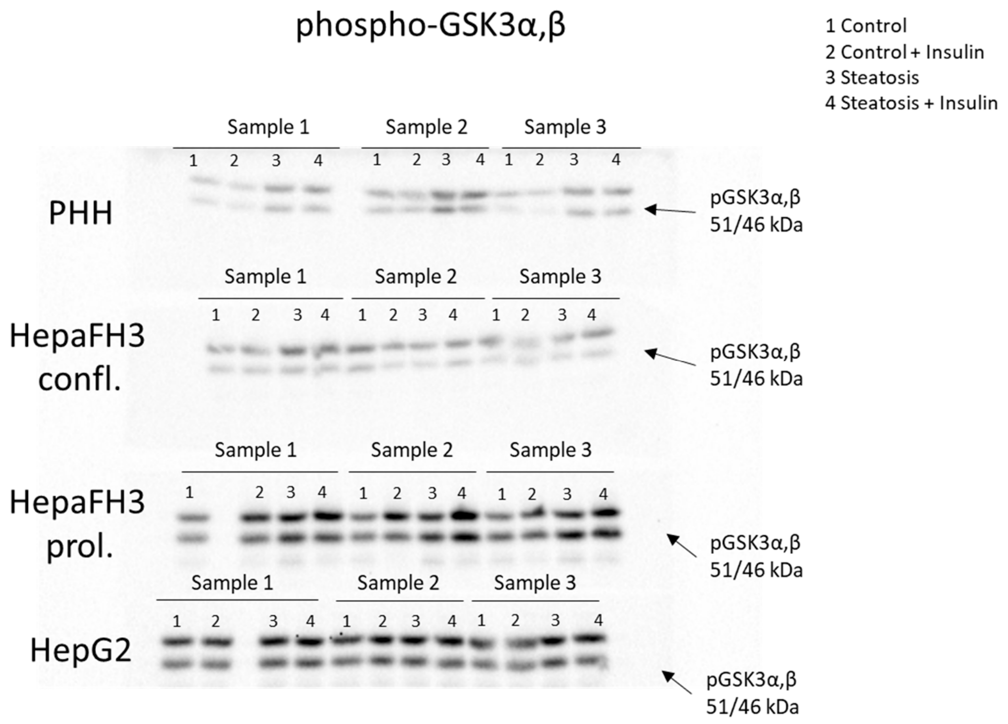

B

		densitometric readings pGSKα,β			
		**PHH**<br>DR-Blank	**HepaFH3 confl.**<br>DR-Blank	**HepaFH3 prol.**<br>DR -Blank	**HepG2**<br>DR - Blank
Sample 1	1	498180	952000	832176	1214568
	2	448860	1061970	1287872	1285112
	3	1193947,5	1620626	1488616	1427584
	4	1123357,5	1473507	1677568	1465552
Sample 2	1	879825	1529955	862016	1261984
	2	1037542,5	853951	1295192	1295280
	3	2066775	824355	1272296	1375008
	4	1797592,5	1080527	2079552	1413944
Sample 3	1	679575	902132	962096	1042984
	2	376950	970375	1178064	1180664
	3	1312117,5	708064	1539816	1441896
	4	1196100	1275232	1690936	1366936

Figure S4 = Western Blot analysis of pGSKα,β (51/46 kDa) for PHHs, HepaFH3 confl., HepaFH3 prol. and HepG2 cells. (A) Cell lysates of the different cell types have been separated by SDS-PAGE and blotted on separate membranes. For all cell types, experiments have been performed in triplicates. **(B)** Densitometric readings (DR) for every band is depicted. Blank of an empty line has been deducted from the DR. Exposure time = 141.4 sec for PHHs, 141.4 sec for HepaFH3 confl., 50.3 sec for HepaFH3 prol., 50.3 sec for HepG2. PageRuler™ Prestained Protein Ladder, 10-180 kDa from Thermo Fisher was used as protein ladder. Confl. = confluent, Prol. = proliferating, DR = densitometric reading.

A

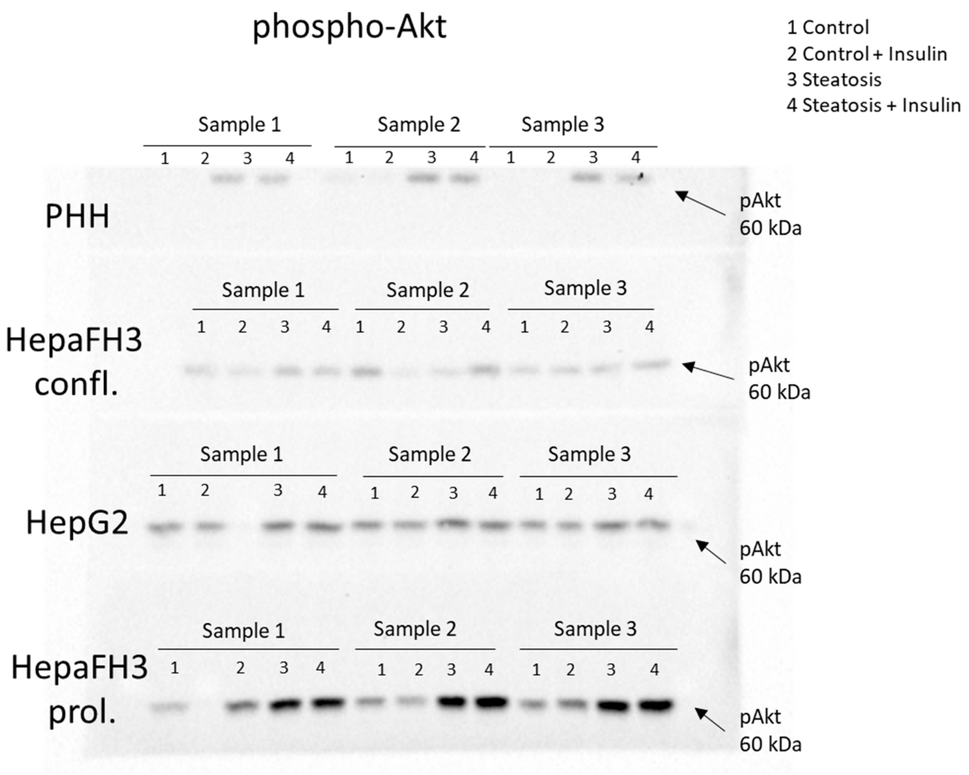

B

		densitometric readings pAkt			
		**PHH**<br>DR-Blank	**HepaFH3 confl.**<br>DR-Blank	**HepaFH3 prol.**<br>DR -Blank	**HepG2**<br>DR - Blank
Sample 1	1	0	1134140	325935	325935
	2	0	682696	903825	903825
	3	1479422	1382388	1747080	1747080
	4	1268274	1185282	1798095	1798095
Sample 2	1	292712	2055564	568035	568035
	2	139020	387268	430545	430545
	3	2347254	746788	1930155	1930155
	4	1926582	2073988	2473500	2473500
Sample 3	1	0	877086	579390	579390
	2	0	991662	832470	832470
	3	2163182	1031814	2177550	2177550
	4	1673000	1431024	2415315	2415315

Figure S5= Western Blot analysis of pAkt (60 kDa) for PHHs, HepaFH3 confl., HepaFH3 prol. and HepG2 cells. **(A)** Cell lysates of the different cell types have been separated by SDS-PAGE and blotted on separate membranes. For all cell types, experiments have been performed in triplicates. **(B)** Densitometric readings (DR) for every band is depicted. Blank of an empty line has been deducted from the DR. Exposure time = 266.2 sec for PHHs, 266.2 sec for HepaFH3 confl., 55.2 sec for HepaFH3 prol., 55.2 sec for HepG2. *PageRuler™ Prestained Protein Ladder, 10-180 kDa from Thermo Fisher was used as protein ladder.* Confl. = confluent, Prol. = proliferating, DR = densitometric reading.

A

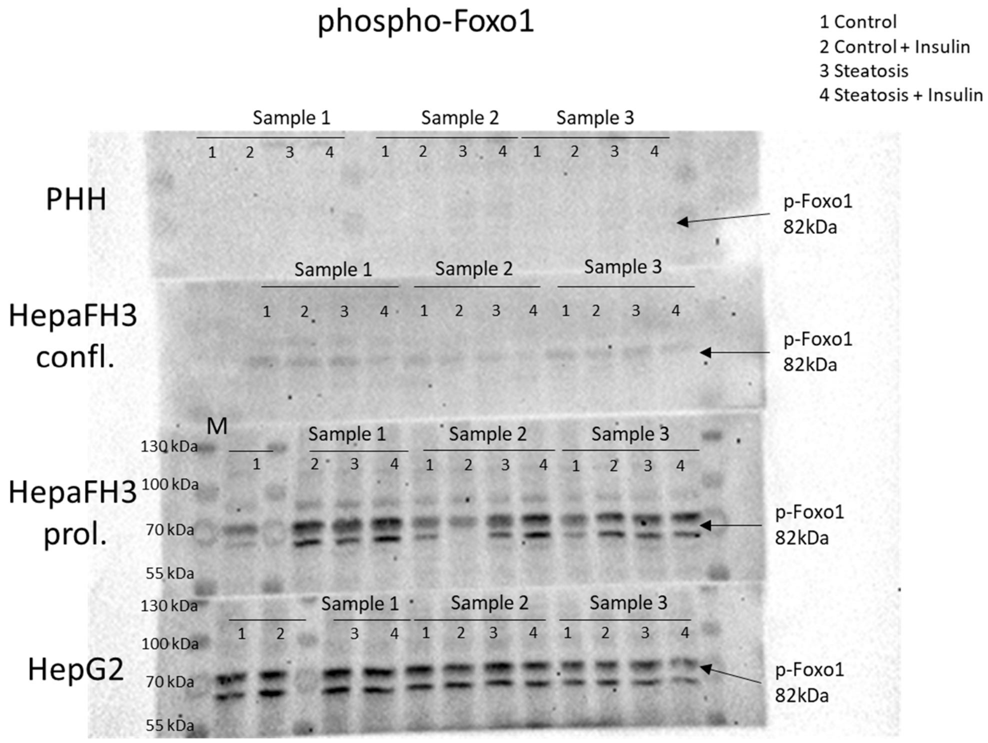

B

		densitometric readings pFoxo1			
		**PHH**	**HepaFH3 confl.**	**HepaFH3 prol.**	**HepG2**
		DR-Blank	DR-Blank	DR -Blank	DR -Blank
Sample 1	1	13286	203775	505980	636847,5
	2	0	178425	1012845	924442,5
	3	27174	184020	830557,5	717727,5
	4	9254	24465	1145902,5	941692,5
Sample 2	1	0	15030	569430	667890
	2	0	49920	291510	577702,5
	3	48132	0	795037,5	764977,5
	4	149548	0	819870	538117,5
Sample 3	1	0	189120	541380	427455
	2	0	187740	559755	476587,5
	3	291004	195285	580335	488175
	4	98476	52020	798082,5	329692,5

Figure S6= Western Blot analysis of pFoxo1 (82 kDa) for PHHs, HepaFH3 confl., HepaFH3 prol. and HepG2 cells. (A) Cell lysates of the different cell types have been separated by SDS-PAGE and blotted on separate membranes. For all cell types, experiments have been performed in triplicates. **(B)** Densitometric readings (DR) for every band is depicted. Blank of an empty line has been deducted from the DR. Exposure time = 600 sec for all blots. PageRuler™ Prestained Protein Ladder, 10-180 kDa from Thermo Fisher was used as protein ladder. Confl. = confluent, Prol. = proliferating, DR = densitometric reading, M = marker.
